# Synthesis and Structure-Activity Relationships of (−)-*cis*-*N*-Normetazocine-Based LP1 Derivatives

**DOI:** 10.3390/ph11020040

**Published:** 2018-05-05

**Authors:** Lorella Pasquinucci, Carmela Parenti, Emanuele Amata, Zafiroula Georgoussi, Paschalina Pallaki, Valeria Camarda, Girolamo Calò, Emanuela Arena, Lucia Montenegro, Rita Turnaturi

**Affiliations:** 1 Department of Drug Sciences, Medicinal Chemistry Section, University of Catania, Viale A. Doria 6, 95125 Catania, Italy; lpasquin@unict.it (L.P.); eamata@unict.it or emanuela.arena@unict.it (E.A.); 2 Department of Drug Sciences, Pharmacology and Toxicology Section, University of Catania, Viale A. Doria 6, 95125 Catania, Italy; cparenti@unict.it; 3 Laboratory of Cellular Signaling and Molecular Pharmacology, Institute of Biosciences and Applications, National Centre for Scientific Research “Demokritos” Agia Paraskevi-Attikis, 15310 Athens, Greece; iro@bio.demokritos.gr (Z.G.); ppallaki@bio.demokritos.gr (P.P.); 4 Department of Medical Sciences, Section of Pharmacology, University of Ferrara, via Fossato di Mortara 19, 44121 Ferrara, Italy; v.camarda@unife.it (V.C.); clg@unife.it (G.C.); 5 Department of Drug Sciences, Pharmaceutical Technology Section, University of Catania, Viale A. Doria 6, 95125 Catania, Italy; lmontene@unict.it

**Keywords:** opioid receptors, radioligand binding, calcium mobilization, benzomorphan

## Abstract

(−)-*cis*-*N*-Normetazocine represents a rigid scaffold able to mimic the tyramine moiety of endogenous opioid peptides, and the introduction of different *N*-substituents influences affinity and efficacy of respective ligands at MOR (mu opioid receptor), DOR (delta opioid receptor), and KOR (kappa opioid receptor). We have previously identified LP1, a MOR/DOR multitarget opioid ligand, with an *N*-phenylpropanamido substituent linked to (−)-*cis*-*N*-Normetazocine scaffold. Herein, we report the synthesis, competition binding and calcium mobilization assays of new compounds **10**–**16** that differ from LP1 by the nature of the *N*-substituent. In radioligand binding experiments, the compounds **10**–**13**, featured by an electron-withdrawing or electron-donating group in the para position of phenyl ring, displayed improved affinity for KOR (K_i_ = 0.85–4.80 μM) in comparison to LP1 (7.5 μM). On the contrary, their MOR and DOR affinities were worse (K_i_ = 0.18–0.28 μM and K_i_ = 0.38–1.10 μM, respectively) with respect to LP1 values (K_i_ = 0.049 and 0.033 μM). Analogous trends was recorded for the compounds **14**–**16**, featured by indoline, tetrahydroquinoline, and diphenylamine functionalities in the *N*-substituent. In calcium mobilization assays, the compound **10** with a *p*-fluorophenyl in the *N*-substituent shared the functional profile of LP1 (pEC_50_^MOR^ = 7.01), although it was less active. Moreover, the *p*-methyl- (**11**) and *p*-cyano- (**12**) substituted compounds resulted in MOR partial agonists and DOR/KOR antagonists. By contrast, the derivatives **13**–**15** resulted as MOR antagonists, and the derivative **16** as a MOR/KOR antagonist (pK_B_^MOR^ = 6.12 and pK_B_^KOR^ = 6.11). Collectively, these data corroborated the critical role of the *N*-substituent in (−)-*cis*-*N*-Normetazocine scaffold. Thus, the new synthesized compounds could represent a template to achieve a specific agonist, antagonist, or mixed agonist/antagonist functional profile.

## 1. Introduction

Natural, semi-synthetic, and synthetic opioid ligands [1,2,3,4], featured with different structural scaffolds, exert their action following recognition of three different opioid receptors, namely mu, delta, and kappa opioid receptors (MOR, DOR, and KOR, respectively), which are members of the large superfamily of G protein coupled receptors (GPCRs) [5] that couple to Gi/o members and other proteins to regulate signaling. Endogenous ligands of these receptors are opioid peptides generally characterized by the highly conserved *N*-terminal tetrapeptidic sequence Tyr–Gly–Gly–Phe [6], although the MOR-selective endogenous peptides, endomorphin-1 (EM-1, Tyr–Pro–Trp–Phe–NH_2_) and endomorphin-2 (EM-2, Tyr–Pro–Phe–Phe–NH_2_), had a different sequence [7]. (−)-*cis*-*N*-Normetazocine, resulting from a progressive simplification of morphine skeleton, represents a rigid scaffold able to support the phenolic ring and the basic nitrogen in a conformation mimicking the tyramine moiety of opioid peptides [8]. In several investigations, the pivotal role of the substituent at the basic nitrogen has emerged [9,10,11,12,13]. Indeed, the *N*-substituent nature influences affinity, selectivity, and activity towards all three opioid receptor subtypes.

**Figure 1:**
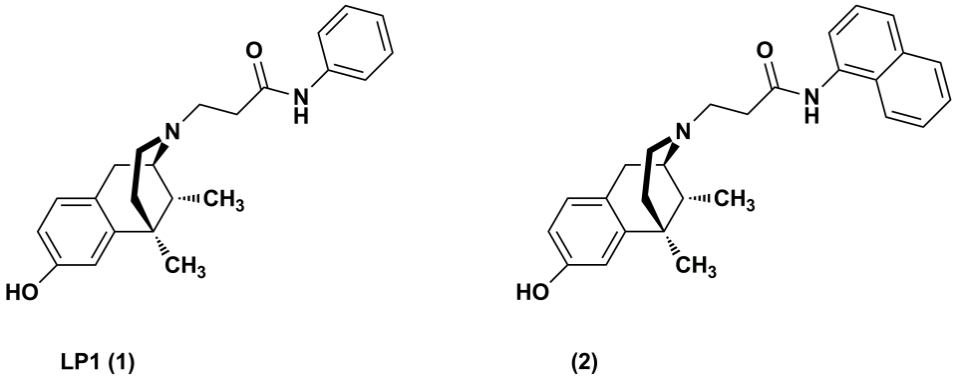
LP1 (**1**) and its *N*-analogue (**2**) structures.

We have previously reported the synthesis and pharmacological characterization of different series of (−)-*cis*-*N*-normetazocine-based ligands [14]. These data demonstrated that the introduction of the acetamido spacer in the basic nitrogen was detrimental for MOR, DOR, and KOR binding. By contrast, the *N*-propanamido spacer elongation provided derivatives with an improved binding profile. Among them, LP1 ((**1**) (3-[(2*R*,6*R*,11*R*)-8-hydroxy-6,11-dimethyl-1,4,5,6-tetrahydro-2,6-methano-3-benzazocin-3(2H)-yl]-*N*-phenylpropanamide, Figure 1), featured with a *N*-phenylpropanamido substituent, resulted in high MOR and significant DOR binding coupled to a MOR/DOR agonist multitarget profile, consistent with its significant antinociceptive effect in nociceptive and persistent pain rat models [15,16,17]. It has also highlighted the importance in the *N*-substituent of a secondary amide for the opioid receptor interactions [14], while the presence of a tertiary amide led to poor K_i_ values, mainly towards MOR. Moreover, the critical role of the aromatic ring in the *N*-phenylpropanamido spacer was assessed. Saturated chains made worse the receptor binding interaction, although the cyclohexyl ring retained a significant MOR affinity [14]. Bulkier aromatic/heteroaromatic rings also resulted in a loss of opioid binding affinity, with the exception of the compound bearing 1-naphthyl ring in the *N*-propanamido chain ((**2**), Figure 1) that retained MOR affinity with a high degree of selectivity. However, this bulkier substituent switched the efficacy profile from agonism to antagonism [18]. The presence of a second positive charge, as well as the shortening of the *N*-substituent spacer, obtained through the insertion of secondary and tertiary ethylamino or propylamino side chains, led to benzomorphan-based compounds able to address the ligand–opioid receptor interaction, mainly at MOR and KOR [19]. Recently, we have also reported the insertion at the basic nitrogen of a shorter and more flexible ethyl spacer with H-bonding groups at carbon 2 as a successful strategy to improve MOR and DOR affinity [20].

**Figure 2:**
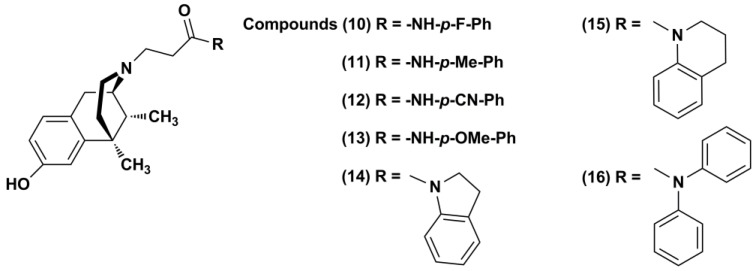
LP1 *N*-derivatives **10–16** structures.

In this study, we extended our structure–activity relationships (SARs) [14,18,19,20] and evaluated the effects of *para* electron-donating or -withdrawing groups insertion in the LP1 phenyl ring (**10**–**13**, Figure 2). Moreover, the employment of phenyl ring surrogates with an increased steric hindrance (using indoline, tetrahydroquinoline, and diphenylamine functionalities) in the *N*-substituent (**14**–**16**, Figure 2) was also assessed. To determine the opioid functional profile, derivatives **10**–**16** were tested by radioligand competition binding and calcium mobilization assays for MOR, DOR, and KOR. Collected data corroborated the importance of the phenyl ring in the *N*-propanamido substituent of the (−)-*cis*-*N*-Normetazocine scaffold for MOR and DOR functional profile. Although all synthesized compounds, in comparison to the lead compound LP1, exhibited a worse efficacy profile, they could represent a template in the achievement of a specific functional profile, by modifying *N*-substituent electronic and steric features.

## 2. Results and Discussion

### 2.1. Chemistry

**Scheme 1:**
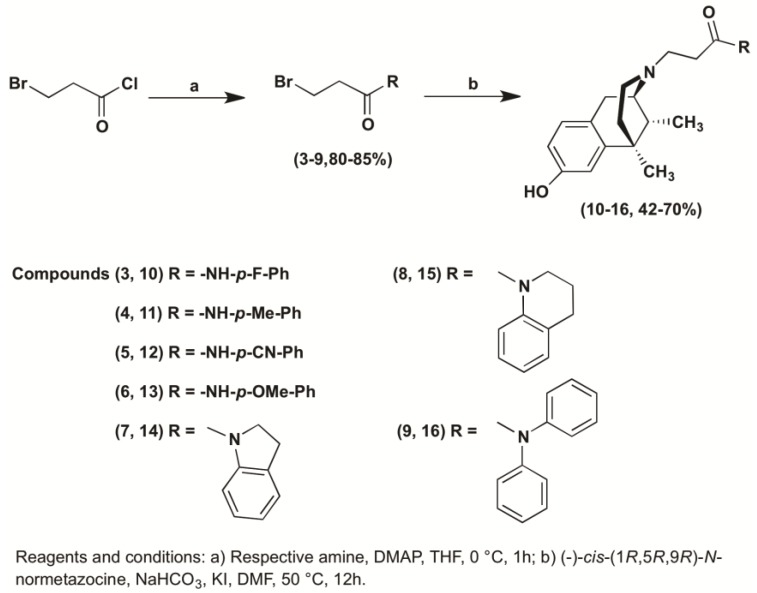
Synthetic pathway.

In Scheme 1, the synthesis of the novel compounds is illustrated. Resolution of (±)-*cis*-*N*-Normetazocine was carried as previously reported [18,21]. Briefly, amides **3**–**9** were prepared by acylation of the respective amines with 3-bromopropionyl chloride in anhydrous THF in argon atmosphere. The target compounds **10**–**16** were obtained by alkylation of (˗)-*cis*-(1*R*,5*R*,9*R*)-*N*-Normetazocine with the respective amides **3**–**9** in DMF. All the synthesized compounds were characterized by IR, ^1^H NMR, ^13^C NMR, mass spectroscopy, and elemental analysis.

### 2.2. Radioligand Binding Assay

All synthesized compounds were tested in vitro by radioligand competition binding using [^3^H]-diprenorphine ([^3^H]-DPN) to measure the affinity for opioid receptor subtypes in membranes from HEK293 cells stably expressing either MOR, DOR, or KOR, as previously described [22,23]. Calculated K_i_ values are listed in Table 1. In comparison to the lead LP1, compounds **10**–**16** displayed a decreased binding affinity for MOR and DOR, while affinity for KOR was increased.

MOR K_i_ values for the *p*-substituted phenyl derivatives **10–13** were six-, five-, four-, and four-times higher than LP1 K_i_ values. Increased K_i_ values at DOR were also recorded for compounds **10–13**. In fact, DOR K_i_ values for compounds **10–13** were twenty-eight-, twelve-, eleven-, and thirty-times higher than LP1 K_i_ values. On the other hand, the KOR affinity of *p*-substituted phenyl analogues was increased. An analogous trend in receptor affinity was also reported for compounds **14–16** that displayed eight-, seven-, and three-times decreased affinity versus MOR and DOR. Notably, the KOR affinity of compounds **14–16** significantly increased, with K_i_ values approximately five-, thirty-seven-, and thirty-seven-times lower than the LP1 K_i_ value. These data once more underline the critical role of *N*-substituent in (−)-*cis*-*N*-Normetazocine scaffold for opioid receptor binding affinity.

Tested compounds **10–16** differ from the LP1 structure by their *N*-substituent nature. In compounds **10–13**, the introduction of an electron-withdrawing or electron-donating group *para* to the phenyl ring in the *N*-phenylpropanamido chain is critical for MOR and DOR interaction, but led to an improved KOR interaction. In compounds **14–16**, the phenyl ring replacement with indoline, tetrahydroquinoline, and diphenylamine functionalities was detrimental for MOR, and mainly for DOR interaction, while the KOR interaction was improved. Thus, the introduction at the *N*-substituent of a phenyl ring with different electronic and steric features significantly improve KOR interaction, but not MOR and DOR. 

### 2.3. Calcium Mobilization Assay

The pharmacological characterization of LP1 derivatives **2** and **10**–**16** was performed by calcium mobilization assay (Table 2). This procedure was preliminarily validated through a series of experiments where the effects of DPDPE, dermorphin, and dynorphin A were assessed in CHO cells expressing the human recombinant opioid receptors and Gα_qi5_ or Gα_qG66Di5_ chimeric proteins [24]. The obtained results highlighted that all the standard compounds tested showed the expected functional profile.

In CHO^MOR^ cells stably expressing the Gα_qi5_ chimeric protein, LP1, **10**, **11**, and **12** were able to stimulate calcium mobilization in a concentration-dependent manner, with LP1 showing the highest potency (pEC_50_ = 7.01 and α = 0.40 ± 0.020), thus resulting in a full MOR agonist, as reported elsewhere [14,15,16]. Compounds **10**, **11**, and **12** were, in fact, approximately ten-times less potent than LP1, and their estimated maximal effects were lower (α = 0.30 ± 0.015, 0.23 ± 0.011, 0.34 ± 0.017, respectively). In cells expressing the Gα_qi5_ chimeric protein, but not MOR, these compounds were found completely inactive up to 10 μM (data not shown). These results collectively demonstrated that compounds **10**, **11**, and **12** behave as MOR partial agonists.

LP1 derivatives **13–16** were not able to stimulate calcium mobilization in CHO^MOR^ cells stably expressing the Gα_qi5_ protein. In antagonist type experiments, compounds **13–16** were capable of inhibiting the stimulatory effects induced by dermorphin, with an antagonist potency range of 5.71–6.30.

In CHO^DOR^ cells stably expressing the Gα_qG66Di5_ chimeric protein, the compound LP1 was able to stimulate calcium mobilization in a concentration-dependent manner, with a pEC_50_ of 5.95 and low maximal effect (concentration response curve was not completed) resulting in a partial DOR agonist. The LP1 functional profile at DOR, previously in vitro detected [14,16] by adenylyl cyclase, and the [^35^S]GTPγS binding assays was controversial. Indeed, LP1 dose-dependently inhibited the cAMP accumulation, however, it was approximately 85-fold less potent than DPDPE. These results suggested that LP1 was a weak DOR agonist [14]. Contrarily, low concentrations (10 nM) of LP1 resulted in 30% inhibition of [^35^S]GTPγS binding [16], suggesting that LP1 at low concentrations acts as an inverse agonist, and inhibits the constitutive coupling that occurs between DOR and G proteins. Similar findings with DOR spontaneous activity and effects of DOR antagonists have been previously described [25]. This effect was reversed at higher LP1 concentrations [16]. These [^35^S]GTPγS binding results reveal the role of LP1 as a DOR antagonist ligand. Based on the fact that LP1 displays a weak effect on adenyl cyclase activity induced in DOR-expressing cells, we suggested that LP1 is a potent DOR antagonist displaying inverse agonist activity. The DOR antagonist profile of LP1 was also detected in vivo by rat tail flick test. In cells expressing the Gα_qG66Di5_ chimeric protein, but not DOR, LP1 was found completely inactive up to 10 μM (data not shown). Compound **10** was able to stimulate calcium mobilization, but only at the higher concentration tested (10 μM). The remaining LP1 derivatives **11–16** were not able to stimulate calcium mobilization in CHO^DOR^ cells stably expressing the Gα_qG66Di5_ protein. In antagonist type experiments, derivatives **11–12** were able to inhibit the stimulatory effects induced by DPDPE, however, the inhibition response curves to these compounds were incomplete. Compounds **13–16** were not able to inhibit the stimulatory effects induced by DPDPE.

In CHO^KOR^ cells stably expressing the Gα_qi5_ chimeric protein, LP1 and most of its derivatives at higher concentration, up to 10 μM, did not stimulate calcium release (data not shown). Compound **15** was able to stimulate calcium mobilization in a concentration-dependent manner with a pEC_50_ of 6.59, behaving as a low potency and low efficacy (α = 0.32 ± 0.016) partial agonist of KOR. In antagonist type experiments, compounds **11**, **12**, and **16** were able to inhibit the stimulatory effects induced by dynorphin A; however, the inhibition response curves to these compounds were incomplete.

In this series, only compound **10** shares a functional profile with LP1, although it was less active, and it binds MOR and DOR with a K_i_ in the micromolar range. *p*-Methyl- (**11**) and *p*-cyano- (**12**) derivatives display comparable functional profile, resulting in MOR partial agonists and DOR/KOR antagonists. Their MOR potencies were higher than compound **10**, but lower than LP1. These results enabled us to recognize how the presence of bulkier substituents and their electronic effect in the *N*-phenylpropanamido chain could be a stringent feature for opioid receptors efficacy profile. The steric hindrance of *N*-substituents in derivatives **13–16** affects the shift from MOR agonism to antagonism. Moreover, in conformationally restrained compounds **14** and **15**, the restriction in the degrees of freedom due to cyclization led to a loss of MOR agonist activity in favor to an antagonist profile. In a previous investigation [18], we already reported that increased ring sizes other than aromatic/heteroaromatic ring orientation of the *N*-substituents with respect to the (−)-*cis*-*N*-Normetazocine scaffold modulated affinity, selectivity, and activity towards MOR. The switching of the functional profile from agonism to antagonism versus MOR (pK_B_ = 7.90 ± 0.60) of the *N*-1-naphthyl derivative (2) was also confirmed in the calcium mobilization assays.

## 3. Materials and Methods

### 3.1. Chemistry

#### 3.1.1. General Experimental Procedures

All commercial chemicals were purchased from Sigma-Aldrich (St. Louis, MO, USA) or Merck (Darmstadt, Germany) and were used without further purification. (±)-*cis*-*N*-Normetazocine was obtained from Fabbrica Italiana Sintetici (Milano, Italy). Melting points were determined in open capillary tubes with a Büchi 530 apparatus (Büchi, Flawil, Switzerland) and are uncorrected. Analytical TLC was performed on silica gel 60 F254 aluminum sheets (Merck, Kenilworth, NJ, USA) with fluorescent indicator. Components were visualized by UV light (λ = 254 nm) and iodine vapors. Flash column chromatography was carried out on Merck silica gel 60 (230–400 mesh). Optical rotations were determined in MeOH solution with a Perkin-Elmer 241 polarimeter (Llantrisant, UK). Infrared spectra were recorded on a 1600 FT-IR Perkin-Elmer instrument (PerkinElmer, Milano, Italy). ^1^H and ^13^C NMR spectra were routinely recorded on a Varian Inova-500 spectrometer in CDCl_3_ or DMSO solution (Varian, Palo Alto, CA, USA); chemical shifts δ are expressed in ppm with reference to tetramethylsilane as an internal standard. GCMS analysis was recorded using a Shimadzu QP500 EI 171 (70 eV) (Shimadzu Scientific Instruments, Columbia, MD, USA). Elemental analyses (C, H, N) were performed on a Carlo Erba 1106 analyzer (Milan, Italy) and the results were within ±0.4% of the theoretical values. All reported compounds had a purity of at least 95%.

#### 3.1.2. General Procedure for the Preparation of 3-Bromo Propanamide Derivatives (**3**–**9**)

A solution of the appropriate amine (1 equiv) and 4-(dimethylamino)pyridine (DMAP) (0.47 equiv) in dry THF (10 mL) was added dropwise to a solution of 3-bromopropionyl chloride (1.5 equiv) in THF (10 mL) cooled to 0 °C. The reaction mixture was kept under vigorous stirring at room temperature (rt) for 1 h, and was then quenched with H_2_O and extracted with CHCl_3_. The organic phase was washed with a saturated aqueous solution of NaHCO_3_ and brine, and dried over anhydrous Na_2_SO_4_. After the solvent was removed in vacuo, the crude product was purified by flash chromatography (cyclohexane/ethyl acetate, 80:20 *v*/*v*) to give compounds **3**–**9**.

*3-Bromo-N-(4-fluorophenyl)propanamide* (**3**). White solid (76%); mp: 146.5–147.5 °C; IR (KBr) νmax 1665 cm^−1^; ^1^H NMR (DMSO, 500 MHz): δ 10.08 (1H, s, exchangeable in D_2_O), 7.62–7.58 (2H, m), 7.15–7.10 (2H, m), 3.71 (2H, t, *J* = 6.5 Hz), 2.92 (2H, t, *J* = 6.5 Hz); ^13^C NMR (DMSO, 125 MHz) δ 168.01, 158.91 (*J* = 238.25 Hz), 135.32, 120.83 (*J* = 7.75 Hz), 115.34 (*J* = 22.37 Hz), 39.29, 29.11; MS (EI) *m*/*z* (%) 244.9 [M + H]^+^, 246.9 [M + H]^+^.

*3-bromo-N-(4-methylphenyl)propanamide* (**4**). White solid (75%); mp: 140.0–140.6 °C; IR (KBr) νmax 1664 cm^−1^; ^1^H NMR (DMSO, 500 MHz) δ 9.89 (1H, s, exchangeable in D_2_O), 7.40 (2H, d, *J* = 8.4 Hz), 7.01 (2H, d, *J* = 8.4 Hz), 3.63 (2H, t, *J* = 6.2 Hz), 2.83 (2H, t, *J* = 6.2 Hz), 2.15 (3H, s); ^13^C NMR (DMSO, 125 MHz) δ 173.24, 141.85, 137.53, 134.36, 124.36, 44.70, 34.69, 25.80; MS (EI) *m*/*z* (%) 241.0 [M + H]^+^, 243.0 [M + H]^+^.

*3-Bromo-N-(4-cyanophenyl)propanamide* (**5**). White solid (85%); mp: 194.5–195.6 °C; IR (KBr) νmax 2222, 1669 cm^−1^; ^1^H NMR (DMSO, 500 MHz) δ 10.47 (1H, s, exchangeable in D_2_O), 7.78–7.75 (4H, m), 3.72 (2H, t, *J* = 6.5 Hz), 2.99 (2H, t, *J* = 6.5 Hz); ^13^C NMR (DMSO, 125 MHz) δ 169.05, 143.03, 133.27, 119.08, 118.97, 105.06, 39.67, 28.68; MS (EI) *m*/*z* (%) 251.9 [M + H]^+^, 253.9 [M + H]^+^.

*3-Bromo-N-(4-methoxyphenyl)propanamide* (**6**). Yellow solid (70%); mp: 107.5–108.9 °C; IR (KBr) νmax 1650 cm^−1^; ^1^H NMR (DMSO, 500 MHz) δ 9.88 (1H, s, exchangeable in D_2_O), 7.49 (2H, d, *J* = 9 Hz), 6.86 (2H, d, *J* = 9 Hz), 3.71 (2H, t, *J* = 6.5 Hz), 3.70 (3H, s), 2.89 (2H, t, *J* = 6.5 Hz); ^13^C NMR (DMSO, 125 MHz) δ 167.54, 155.19, 132.11, 120.60, 113.81, 55.11, 39.24, 29.31; MS (EI) *m*/*z* (%) 257.0 [M + H]^+^, 259.0 [M + H]^+^.

*3-Bromo-1-(2,3-dihydro-1H-indol-1-yl)propan-1-one* (**7**). White solid (79%); mp: 91.0–91.8 °C; IR (KBr) νmax 1653 cm^−1^; ^1^H NMR (CDCl_3_, 500 MHz) δ 8.22 (1H, d), 7.21–7.18 (2H, m), 7.04–7.01 (1H, t), 4.06 (2H, t, *J* = 6.8 Hz), 3.73 (2H, t, *J* = 6.6 Hz), 3.21 (2H, t, *J* = 6.8 Hz), 3.02 (2H, t, *J* = 6.6 Hz); ^13^C NMR (CDCl_3_, 125 MHz) δ 168.74, 142.41, 131.43, 126.91, 124.50, 123.01, 116.83, 48.53, 38.60, 31.00, 27.88; MS (EI) *m*/*z* (%) 253.0 [M + H]^+^, 255.0 [M + H]^+^.^.^

*3-Bromo-1-(3,4-dihydroquinolin-1(2H)-yl)propan-1-one* (**8**). White solid (70%); mp: 67.5–68.5 °C; IR (KBr,) νmax 1652 cm^−1^; ^1^H NMR (CDCl_3_, 500 MHz) δ 7.21–7.10 (4H, m), 3.81 (2H, t, *J* = 6.6 Hz), 3.67 (2H, t, *J* = 6.8 Hz), 3.08 (2H, t, *J* = 6.8 Hz), 2.72 (2H, t, *J* = 6.6 Hz), 1.97 (2H, q, *J* = 6.6 Hz); ^13^C NMR (CDCl_3_, 125 MHz) δ 169.84, 138.68, 128.78, 127.67, 126,41, 125.86, 124.74, 43.35, 37.70, 28.27, 26.88, 24.22; MS (EI) *m*/*z* (%) 267.0 [M + H]^+^, 269.0 [M + H]^+^.

*3-Bromo-N,N-diphenylpropanamide* (**9**). White solid (72%); mp: 95.9–96.4 °C; IR (KBr,) νmax 1673 cm^−1^; ^1^H NMR (CDCl_3_, 500 MHz) δ 7.28–7.21 (10H, m), 3.65 (2H, t, *J* = 6.6 Hz), 2.83 (2H, t, *J* = 6.6 Hz); ^13^C NMR (CDCl_3_, 125 MHz) δ 169.15, 142.51, 128.86, 126.13, 125.00, 38.12, 27.57; MS (EI) *m*/*z* (%) 303.0 [M + H]^+^, 305.0 [M + H]^+^.

#### 3.1.3. General Procedure for the Synthesis of N-Substituted (−)-Cis-N-Normetazocine Derivatives (**10**–**16**)

A mixture of (˗)-cis-(1R,5R,9R)-N-Normetazocine (1 equiv), the appropriate 3-bromoamide derivatives (**3**–**9**, 1.5 equiv), NaHCO_3_ (1.5 equiv), and a catalytic amount of KI was stirred in DMF at 50 °C for 12 h. After cooling, the reaction mixture was concentrated under vacuum to remove DMF. The resulting residue was purified by flash chromatography using a silica gel column with a CHCl_3_/CH_3_OH (95:5, v/v) solvent system. Target compounds **10**–**16** were recrystallized from absolute ethanol to give white solids.

*N-(4-Fluorophenyl)-3-[(2R,6R,11R)-8-hydroxy-6,11-dimethyl-1,4,5,6-tetrahydro-2,6-methano-3-benzazocin-3(2H)-yl]propanamide* (**10**). White solid (50%); mp: 199.7–201.3 °C; ${\left[\alpha \right]}_{D}^{25}$= −56° (c 1.0, MeOH); ^1^H NMR (DMSO, 500 MHz) δ 10.23 (1H, s), 7.62 (2H, d, *J* = 8.8 Hz), 7.15 (2H, d, *J* = 8.8 Hz), 6.89 (1H, d, *J* = 8.4 Hz), 6.59 (1H, d, *J* = 2.5 Hz), 6.53 (1H, dd, *J* = 8.4 Hz, *J* = 2.5 Hz), 2.91–2.71 (3H, m), 2.62–2.44 (6H, m), 1.99–1.95 (1H, m), 1.72–1.71 (1H, m), 1.27 (3H, s), 1.20 (1H, m), 0.78 (3H, d, *J* = 5 Hz); ^13^C NMR (DMSO, 125 MHz) δ 170.18, 158.73 (*J* = 240.37 Hz), 155.43, 142.28, 135.63, 127.76, 126.34, 120.70 (*J* = 7.75 Hz), 115.30 (*J* = 21.87 Hz), 112.87, 111.80, 56.88, 50.37, 44.80, 41.84, 41.26, 35.88, 34.04, 25.24, 23.20, 13.89; MS (EI) *m*/*z* (%) 383.2 [M + H]^+^, 229.9 [M-CH_2_CONHC_6_H_4_F]; anal. C, 72.11; H, 7.19; N, 7.22%, calcd for C_23_H_27_FN_2_O_2_ (382,471) C, 72.23; H, 7.12; N, 7.32%.

*3-[(2R,6R,11R)-8-Hydroxy-6,11-dimethyl-1,4,5,6-tetrahydro-2,6-methano-3-benzazocin-3(2H)-yl]-N-(4-methylphenyl)propanamide* (**11**). White solid (61%); mp: 180.5–181.8 °C; ${\left[\alpha \right]}_{D}^{25}$= −51° (c 1.0, MeOH); ^1^H NMR (DMSO, 500 MHz) δ 10.05 (1H, s), 7.44 (2H, d, *J* = 8.0 Hz), 7.08 (2H, d, *J* = 8.0 Hz), 6.86 (1H, d, *J* = 8.5 Hz), 6.60 (1H, d, *J* = 2.5 Hz), 6.50 (1H, dd, *J* = 8.5 Hz, *J* = 2.5 Hz), 2.90 (1H, m), 2.87–2.82 (2H, m), 2.67–2.66 (2H, m), 2.58–2.49 (2H, m), 2.45-2.35 (2H, m), 2.23 (3H, s), 2.00–1.90 (1H, m), 1.692–1.632 (1H, m), 1.25 (3H, s), 1.21 (1H, m), 0.75 (3H, d, *J* = 5 Hz); ^13^C NMR (DMSO, 125 MHz) δ 170.00, 155.44, 142.24, 136.73, 131.80, 129.04, 127.77, 126.30, 118.99, 112.88, 111.80, 56.88, 50.39, 44.80, 41.81, 41.24, 35.87, 34.95, 25.23, 23.19, 20.38, 13.87; MS (EI) *m*/*z* (%) 379.3 [M + H]^+^, 230.2 [M-CH_2_CONHC_7_H_7_]; anal. C, 76.02; H, 8.03; N, 7.22%, calcd for C_24_H_30_N_2_O_2_ (378,507) C, 76.16; H, 7.99; N, 7.40%.

*N-(4-Cyanophenyl)-3-[(2R,6R,11R)-8-hydroxy-6,11-dimethyl-1,4,5,6-tetrahydro-2,6-methano-3-benzazocin-3(2H)-yl]propanamide* (**12**). White solid (67%); mp: 215.1–216 °C; ${\left[\alpha \right]}_{D}^{25}$= −54.5° (c 1.0, MeOH); ^1^H NMR (DMSO, 500 MHz) δ 10.56 (1H, s), 7.76 (4H, m), 6.87 (1H, d, *J* = 8.5 Hz), 6.61 (1H, d, *J* = 2.5 Hz), 6.51 (1H, dd, *J* = 8.5 Hz, *J* = 2.5 Hz), 2.89–2.79 (3H, m), 2.73–2.67 (2H, m), 2.59–2.46 (4H, m), 2.02–1.97 (1H, m), 1.74–1.63 (1H, m), 1.25 (3H, s), 1.22 (1H, m), 0.75 (3H, d, *J* = 5 Hz); ^13^C NMR (DMSO, 125 MHz) δ 171.17, 155.44, 142.79, 142.24, 133.25, 127.76, 126.29, 119.04, 118.96, 112.87, 111.79, 104.67, 56.93, 50.16, 44.81, 41.78, 41.18, 35.85, 35.27, 25.21, 23.25, 13.87; MS (EI) *m*/*z* (%) 390.3 [M + H]^+^, 230.2 [M-CH_2_CONHC_7_H_4_N]; anal. C, 73.89; H, 7.05; N, 10.52%, calcd for C_24_H_27_N_3_O_2_ (389,490) C, 74.01; H, 6.99; N, 10.79%.

*3-[(2R,6R,11R)-8-Hydroxy-6,11-dimethyl-1,4,5,6-tetrahydro-2,6-methano-3-benzazocin-3(2H)-yl]-N-(4-methoxyphenyl)propanamide* (**13**). White solid (58%); mp: 172.9–173.5 °C;$\;{\left[\alpha \right]}_{D}^{25}$= −53° (c 1.0, MeOH); ^1^H NMR (DMSO, 500 MHz) δ 10.01 (s, 1H), 7.47 (2H, d, *J* = 9.0 Hz), 6.87 (3H, dd, *J* = 9.0 Hz, *J* = 8.4 Hz), 6.61 (1H, d, *J* = 2.5 Hz), 6.52 (1H, dd, *J* = 8.5 Hz, *J* = 2.5 Hz), 3.71 (3H, s), 2.90–2.89 (1H, m), 2.83–2.80 (2H, m), 2.73–2.67 (2H, m), 2.59–2.54 (2H, m), 2.41–2.40 (2H, m), 2.01–1.97 (1H, m), 1.75–1.65 (1H, m), 1.26 (3H, s), 1.25–1.23 (1H, m), 0.76 (3H, d, *J* = 5 Hz); ^13^C NMR (DMSO, 125 MHz) δ 169.72, 155.43, 154.98, 142.26, 132.42, 127.76, 126.32, 120.48, 113.81, 112.87, 111.79, 56.86, 55.10, 50.43, 44.78, 41.80, 41.25, 35.87, 34.87, 25.22, 23.15, 13.87; MS (EI) *m*/*z* (%) 395.3 [M + H]^+^, 230.3 [M-CH_2_CONHC_7_H_7_O]; anal. C, 73.00; H, 7.68; N, 7.08%, calcd for C_24_H_27_N_3_O_2_ (394,506) C, 73.07; H, 7.66; N, 7.10%.

*1-(2,3-Dihydro-1H-indol-1-yl)-3-[(2R,6R,11R)-8-hydroxy-6,11-dimethyl-1,4,5,6-tetrahydro-2,6-methano-3-benzazocin-3(2H)-yl]propan-1-one* (**14**). White solid (56%); mp: 169 °C dec; ${\left[\alpha \right]}_{D}^{25}$= −38.5° (c 1.0, MeOH); ^1^H NMR (DMSO, 500 MHz) δ 8.07 (1H, d), 7.22 (1H, d, *J* = 7.4 Hz), 7.14 (1H, t, *J* = 7.8 Hz), 6.98 (1H, t, *J* = 7.4 Hz), 6.89 (1H, d, *J* = 8.4 Hz), 6.61 (1H, d, *J* = 2.2 Hz), 6.52 (1H, dd, *J* = 8.4 Hz, *J* = 2.2 Hz), 4.11 (2H, t, *J* = 8.5 Hz), 3.13 (2H, t, *J* = 8.5 Hz), 2.89–2.81 (3H, m), 2.68–2.64 (2H, m), 2.55–2.50 (4H, m), 2.00–1.98 (1H, m), 1.74–1.64 (1H, m), 1.26 (3H, s), 1.20 (1H, m), 0.76 (3H, d, *J* = 5 Hz); ^13^C NMR (DMSO, 125 MHz) δ 172.30, 153.70, 147.02, 142.50, 134.13, 128.17, 127.52, 126.76, 124.68, 123.64, 116.40, 112.83, 111.76, 57.20, 47.07, 46.71, 46.46, 40.26, 38.50, 35.75, 33.67, 28.21, 25.46, 22.59, 14.52; MS (EI) *m*/*z* (%) 391.5 [M + H]^+^, 230.1 [M-CH_2_CONC_8_H_8_]; anal. C, 76.68; H, 7.90; N, 7.05%, calcd for C_25_H_30_N_2_O_2_ (390,518) C, 76.89; H, 7.74; N, 7.17%.

*1-(3,4-Dihydroquinolin-1(2H)-yl)-3-[(2R,6R,11R)-8-hydroxy-6,11-dimethyl-1,4,5,6-tetrahydro-2,6-methano-3-benzazocin-3(2H)-yl]-propan-1-one* (**15**). White solid (42%); mp: 92.2 °C dec; ${\left[\alpha \right]}_{D}^{25}$= −37° (c 1.0, MeOH); ^1^H NMR (DMSO, 500 MHz) δ 7.37 (1H, d, *J* = 8.0 Hz), 7.19–7.07 (3H, m), 6.82 (1H, d, *J* = 8.2 Hz), 6.56 (1H, d, *J* = 2.4 Hz), 6.47 (1H, dd, *J* = 8.2 Hz, *J* = 2.2 Hz), 3.66 (2H, t, *J* = 6.4 Hz), 2.75–2.67 (5H, m), 2.63–2.46 (4H, m), 2.30–2.26 (2H, m), 1.91–1.84 (2H, m), 1.65–1.56 (2H, m), 1.21 (3H, s), 1.11 (1H, m), 0.69 (3H, d, *J* = 5 Hz); ^13^C NMR (DMSO, 125 MHz) δ 171.25, 155.39, 142.27, 139.10, 138.91, 128.37, 127.72, 126.34, 126.12, 125.68, 124.64, 112.83, 111.76, 57.20, 51.00, 47.01, 45.09, 41.76, 41.14, 35.75, 35.07, 32.67, 26.21, 25.25, 23.68, 23.19, 13.82; MS (EI) *m*/*z* (%) 405.1 [M + H]^+^, 230.1 [M-CH_2_CONC_9_H_10_]; anal. C, 77.03; H, 8.05; N, 6.78%, calcd for C_26_H_32_N_2_O_2_ (404,544) C, 77.19; H, 7.97; N, 6.92%.

*3-[(2R,6R,11R)-8-Hydroxy-6,11-dimethyl-1,4,5,6-tetrahydro-2,6-methano-3-benzazocin-3(2H)-yl]-N,N-diphenylpropanamide* (**16**). White solid (70%); mp: 173.5 °C dec;$\;{\left[\alpha \right]}_{D}^{25}$= −32° (c 1.0, MeOH); ^1^H NMR (DMSO, 500 MHz) δ 7.40–7.32 (10H, m), 6.84 (1H, d, *J* = 8.5 Hz), 6.58 (1H, d, *J* = 2.5 Hz), 6.49 (1H, dd, *J* = 8.4 Hz, *J* = 2.5 Hz), 2.80–2.73 (1H, m), 2.70–2.67 (2H, m), 2.60–2.56 (1H, m), 2.49–2.45 (1H, m), 2.33–2.23 (2H, m), 1.92–1.88 (2H, m), 1.71–1.70 (1H, m), 1.65–1.59 (1H, m), 1.25 (3H, s), 1.15 (1H, m), 0.73 (3H, d, *J* = 5 Hz); ^13^C NMR (DMSO, 125 MHz) δ 171.24, 155.38, 143.04, 142.31, 128.46, 127.71, 126.39, 126.15, 125.00, 112.82, 111.76, 57.27, 50.79, 45.04, 41.76, 41.12, 35.81, 33.42, 25.25, 23.36, 13.87; MS (EI) *m*/*z* (%) 441.4 [M + H]^+^, 230.1 [M-CH_2_CONC_12_H_10_]; anal. C, 79.32; H, 7.54; N, 6.44%, calcd for C_29_H_32_N_2_O_2_ (440,576) C, 79.06; H, 7.32; N, 6.36%.

### 3.2. Biological Assays

#### 3.2.1. Drugs and Reagents

[^3^H]-Diprenorphine ([^3^H]-DPN) (42.30 C_i_/mmol) was purchased from Perkin Elmer Life Sciences (NET11212, 250μCi, Boston, MA, USA) and naloxone hydrochloride was purchased from Sigma-Aldrich (St. Louis, MO, USA). DPDPE ([D-Pen^2^,D-Pen^5^]enkephalin) and dynorphin A were purchased from Neosystem. Dermorphin was prepared and purified in house as previously described [26]. Stock solutions (10 mM) of peptides were made in distilled water, while LP1 and its derivatives in DMSO and kept at −20 °C until use. The compounds were solubilized in saline at 1 μM, with the only exception of naloxone, which was at 10 μM. Successive dilutions of standard ligands were made in saline, whereas LP1 and its analogues were solubilized in HBSS (Hank’s balanced salt solution)/HEPES (4-(2-hydroxyethyl)-1-piperazineethanesulfonic acid) (20 mM) buffer (containing 0.005% bovine serum albumin (BSA) fraction V). Tissue culture media and supplements were from Invitrogen (Carlsbad, CA, USA) and from Cambrex Bioscience (Walkersville, MD, USA).

#### 3.2.2. Cell Membrane Preparations

Confluent monolayers of HEK293 (human embryonic kidney 293) cells stably expressing rat-MOR, rat-DOR, or human-KOR were harvested, collected by centrifugation at 1500 rpm for 5 min, and washed once with phosphate-buffered saline (PBS) at pH 7.5. Membranes from cell pellets were carried out as described by Georgoussi and Zioudrou [25] (1993) and Papakonstantinou et al. [20] (2015). Briefly, cell pellets were resuspended in ice-cold membrane buffer (10 mM Tris-HCl, pH 7.5 and 0.1 mM EDTA), homogenized, and centrifuged at 2,000 g for 3 min at 4 °C. Supernatants were further centrifuged at 45,000 rpm for 30 min at 4 °C. The membrane pellet was resuspended in ice-cold membrane buffer at a protein concentration of approximately 1 mg/mL and stored in aliquots at −70 °C. Protein concentration was determined according to Bradford assay [27,28].

#### 3.2.3. In Vitro Competition Radioligand Binding Assay

In the experiments designed to define compound affinity and selectivity for MOR, DOR, and KOR, membranes expressing each of these opioid receptors (20 mg) were incubated at 30 °C for 45 min in buffer containing 50 mM Tris-HCl, pH 7.5, 100 mM NaCl, and 10 mM MgCl_2_ as described by Georgoussi et al. [29] (1997). The ability of compounds **10**–**16** to displace [^3^H]-DPN (3.75 nM) binding to the opioid receptors was assessed as described by Morou And Georgoussi [30] (2005). Non-specific binding was measured in the presence of 10 mΜ Naloxone. The reaction was stopped by rapid filtration and three washes in ice-cold 50 mM Tris-HCl, pH 7.4, through GF/C filters (Whatman, Maidstone, UK) using an automated cell harvester (Brandel Inc., Gaithersburg, MD, USA). The radioactivity was measured by liquid scintillation counting (Liquid Scintillation Analyzer, Packard). 

#### 3.2.4. Transfection Procedures and Cell Culture

Chinese hamster ovary (CHO) cells expressing the recombinant human MOR and KOR (CHO^MOR^ and CHO^KOR^) were kindly provided by Dr. Larry Toll (SRI International, Menlo Park, CA, USA), and CHO cells expressing the recombinant human DOR (CHO^DOR^) were supplied by Dr. Eva Varga (Department of Medical Pharmacology, The University of Arizona, Tucson, AZ, USA). CHO cells lines permanently coexpressing the opioid receptors and the C-terminally modified Gα_qi5_ were prepared by infecting the CHO lines described above with a recombinant retrovirus expressing the chimeric α subunit and the hygromycin resistance gene. Similarly, CHO cells lines permanently coexpressing DOR and the C-terminally modified Gα_qG66Di5_ were prepared by infecting the CHO lines described above with a recombinant retrovirus expressing the chimeric α subunit and the hygromycin resistance gene. Polyclonal cell lines were generated using the pantropic retroviral expression system form BD-Clontech, as described previously. Stable lines were selected under hygromycin B (100 μg/mL) and geneticin (600 μg/mL) for 2–3 weeks after the infection.

#### 3.2.5. Cell Culture and Calcium Mobilization Experiments

CHO cells, expressing human opioid receptors and the Gα_qi5_ protein, and CHO_DOR_, expressing the Gα_qG66Di5_ protein, were maintained in culture medium consisting of Dulbecco minimum essential medium (DMEM) and Ham F-12 (1:1) (50/50) supplemented with 10% fetal calf serum (FCS), L-glutamine (2 mM), geneticin (200 μg/mL; G418), hygromycin B (100 μg/mL) at 37 °C in 5% CO_2_ humidified air. When confluence was reached (3–4 days), cells were subcultured as required using trypsin/EDTA, and used for experimentation. The different cell lines were seeded at a density of 50,000–40,000 cells/well into black 96-well, clear-bottomed plates. After 24 h incubation, the cells were loaded with medium supplemented with 2.5 mM probenecid, 3 μM of the calcium sensitive fluorescent dye Fluo-4 AM, and 0.01% pluronic acid, for 30 min at 37 °C. Afterwards, the loading solution was aspirated and 100 μL/well of assay buffer—HBSS supplemented with 20 mM HEPES, 2.5 mM probenecid and 500 μM Brilliant Black (Aldrich, St. Louis, MO, USA)—was added. Serial dilutions of ligands for experimental use were made in HBSS/HEPES (20 mM) buffer (containing 0.005% BSA fraction V). After placing both plates (cell culture and compound plate) into the FlexStation II (Molecular Device, Union City, San Jose, CA 94587, USA), fluorescence changes were measured at room temperature. Online additions were carried out in a volume of 50 μL/well. Antagonists were incubated 15 min before the addition of the agonist. Maximum change in fluorescence, expressed in percent of baseline fluorescence, was used to determine agonist response.

#### 3.2.6. Data Analysis

Analysis of the binding data was performed using the Origin 7.5 software (OriginLab Corporation, Northampton, MA, USA). In calcium mobilization experiments, data were analyzed by non-linear curve fitting equation, using GraphPad 5.0 software. For potency values, 95% confidence limits (CL_95%_) were indicated. Ligand efficacy was expressed as intrinsic activity (α), calculated as the ratio between the E_max_ of the ligand and that of the respective standards. Calcium mobilization data were expressed as FIU (fluorescence intensity units) in percent, over the baseline. Agonist potencies are given as pEC_50_ (the negative logarithm to base 10 of the molar concentration of an agonist that produces 50% of the maximal possible effect, E_max_). Concentration response curves to agonists were fitted with the following equation:

$$\mathrm{Effect}\;=\;\mathrm{baseline}\;+\;\left(E_{\max\;}-\;\mathrm{baseline}\right)/\left(1+10^{\left({\mathrm{LogEC}}_{50\;}-\;X\right)\;*\;\mathrm{HillSlope}}\right)$$

where X is the agonist concentration.

Antagonist potencies were derived from inhibition experiments and expressed as pK_B_ calculated from the following equation:

$$K_{B\;}={\;\mathrm{IC}}_{50}/\left({\left[2\;+{\left(\left[A\right]/{\mathrm{EC}}_{50}\right)}^{n}\right]}^{1/n}\right)\;–\;1$$

where IC_50_ is the concentration of antagonist that produces 50% inhibition of the agonist response, [A] is the concentration of agonist, EC_50_ is the concentration of agonist producing a 50% maximal response, and n is the Hill coefficient of the concentration response curve to the agonist.

## 4. Conclusions

Here, to improve the body of information on (−)-*cis*-*N*-Normetazocine-based compounds SARs, the synthesis and in vitro characterization of a new series of compounds (**10**–**16**) were reported. Collectively, our data corroborated the importance of the phenyl ring in the *N*-propanamido substituent of the (−)-*cis*-*N*-Normetazocine scaffold of the reference compound LP1 for MOR and DOR functional profile. Although all synthesized compounds, in comparison to the lead compound LP1, exhibited a worse efficacy profile, they could represent a template in the achievement of a specific functional profile, by modifying *N*-substituent electronic and steric features.

## Figures and Tables

**Table 1:** Opioid receptor binding affinity values of LP1, and its derivatives **2** and **10**–**16**.

Compound	K_i_ (μM) ± SEM ^a,b^		
	MOR	DOR	KOR
**LP1 (1)**	0.049 ± 0.015	0.033 ± 0.020	7.500 ± 0.015
**2** [18]	0.038±0.004	0.210 ± 0.030	0.800 ± 0.016
**10**	0.280 ± 0.013	0.920 ± 0.120	4.800 ± 0.950
**11**	0.240 ± 0.015	0.410 ± 0.023	1.010 ± 0.110
**12**	0.210 ± 0.024	0.380 ± 0.034	0.850 ± 0.180
**13**	0.180 ± 0.011	1.100 ± 0.110	4.000 ± 0.180
**14**	0.400 ± 0.030	1.400 ± 0.120	1.600 ± 0.120
**15**	0.360 ± 0.023	ND^c^	0.200 ± 0.020
**16**	0.170 ± 0.013	ND	0.200 ± 0.030

^a^ Values are means ± SEM of three separate experiments, each carried out in duplicate; ^b^ K_i_ values were obtained as [^3^H]-DPN displacement for MOR, DOR, and KOR; ^c^ ND not determined.

**Table 2:** Effects of LP1 and its derivatives **2** and **10**–**16** in CHO cells expressing Gα_qi5_ or Gα_qG66Di5_ chimeric G protein and recombinant human opioid receptors in the calcium mobilization assay.

Compound	MOR		DOR		KOR	
	pEC_50_ (CL_95%_) ^a^	pK_B_^b^ (CL_95%_)	pEC_50_ (CL_95%_) ^a^	pK_B_^b^ (CL_95%_)	pEC_50_ (CL_95%_) ^a^	pK_B_^b^ (CL_95%_)
**LP1 (1)**	7.01 (6.29–7.73)	ND ^c^	5.95 (5.39–6.51)	ND	crc incomplete ^d^	ND
**2**	Inactive ^e^	7.90 (7.30–8.50)	Inactive	6.78 (6.15–7.41)	Inactive	5.86 (4.89–6.83)
**10**	5.67 (4.82–6.52)	ND	crc incomplete	Inactive	crc incomplete	ND
**11**	5.85 (5.34–6.36)	ND	Inactive	5.83 (5.53–6.13)	Inactive	5.85 (5.65–6.05)
**12**	5.99 (5.46–6.52)	ND	Inactive	5.82 (5.18–6.46)	Inactive	5.90 (5.40–6.40)
**13**	Inactive	6.30 (5.81–6.79)	Inactive	Inactive	Inactive	Inactive
**14**	Inactive	5.94 (5.26–6.62)	Inactive	Inactive	Inactive	Inactive
**15**	Inactive	5.71 (4.63–6.18)	Inactive	Inactive	6.59 (6.35–6.83)	ND
**16**	Inactive	6.12 (5.46–6.78)	Inactive	Inactive	Inactive	6.11 (5.78–6.44)

Data are mean ± SEM of at least five separate experiments. ^a^ For pEC_50_ and pK_B_ values, the 95% confidence limits are given in brackets; ^b^ The antagonistic properties of these compounds were tested using dermorphin, DPDPE, and dynorphin A as agonists; ^c^ ND: not determined; ^d^ incomplete concentration response curves at 10 μM; ^e^ inactive compound at 10 μM.
